# Genome-wide analysis of DNA methylation in obese, lean, and miniature pig breeds

**DOI:** 10.1038/srep30160

**Published:** 2016-07-22

**Authors:** Yalan Yang, Rong Zhou, Yulian Mu, Xinhua Hou, Zhonglin Tang, Kui Li

**Affiliations:** 1 State Key Laboratory of Animal Nutrition, Institute of Animal Science, Chinese Academy of Agricultural Sciences, Beijing 100193, China; 2 Agricultural Genome Institute at Shenzhen, Chinese Academy of Agricultural Sciences, Shenzhen, 518124, China

## Abstract

DNA methylation is a crucial epigenetic modification involved in diverse biological processes. There is significant phenotypic variance between Chinese indigenous and western pig breeds. Here, we surveyed the genome-wide DNA methylation profiles of blood leukocytes from three pig breeds (Tongcheng, Landrace, and Wuzhishan) by methylated DNA immunoprecipitation sequencing. The results showed that DNA methylation was enriched in gene body regions and repetitive sequences. LINE/L1 and SINE/tRNA-Glu were the predominant methylated repeats in pigs. The methylation level in the gene body regions was higher than in the 5′ and 3′ flanking regions of genes. About 15% of CpG islands were methylated in the pig genomes. Additionally, 2,807, 2,969, and 5,547 differentially methylated genes (DMGs) were identified in the Tongcheng vs. Landrace, Tongcheng vs. Wuzhishan, and Landrace vs. Wuzhishan comparisons, respectively. A total of 868 DMGs were shared by the three contrasts. The DMGs were significantly enriched in development- and metabolism-related biological processes and pathways. Finally, we identified 32 candidate DMGs associated with phenotype variance in pigs. Our research provides a DNA methylome resource for pigs and furthers understanding of epigenetically regulated phenotype variance in mammals.

The domestic pig (*Sus scrofa*) is an economically important food source and an attractive disease model because of anatomical, physiological, pathological, and genomic similarities to humans^1^,^2^,^3^. In modern agricultural industry, pigs have undergone strong long-term artificial selection and developed genetic and phenotypic divergence. In comparison with Chinese indigenous pig breeds, such as Wuzhishan (a miniature breed) and Tongcheng pigs (an obese-type breed), Landrace pigs (a western lean pig breed) show much more rapid muscle growth, greater body weight, and a higher percentage of lean meat. In contrast, Tongcheng pigs exhibit low muscle mass and a high body fat percentage. Miniature Wuzhishan pigs are much smaller and lighter than Tongcheng and Landrace pigs and are recognized as an attractive biomedical model (adults weigh < 40 kg)^4^. These phenotypic differences make pigs highly suitable for animal agriculture and comparative studies^5^,^6^.

DNA methylation is one of the most important and stable epigenetic modifications in eukaryotes^7^. DNA methylation plays an important role in many biological process, including gene expression regulation^8^, genomic imprinting^9^, transposon silencing^10^, X chromosome inactivation^11^, and disease development^12^,^13^. In addition, DNA methylation is crucial for maintaining chromatin structure, chromosome stability, and transcription^14^. Multiple approaches have been developed to analyze DNA methylation profiles at the genome-wide level, including bisulfite-sequencing (BS-seq), methylated DNA immunoprecipitation-chip (MeDIP-chip), reduced representation bisulfite sequencing (RRBS), methylated DNA immunoprecipitation-sequencing (MeDIP-seq), and enzyme digestion-based techniques^15^. BS-seq is the gold standard for analyzing DNA methylomes^16^. However, despite its high resolution, BS-seq is expensive and time consuming. RRBS reduces the portion of the genome analyzed through MspI digestion and fragment size selection, but it is less efficient when using tissue samples and requires much deeper sequence coverage^17^. MeDIP-chip is limited by the requirement of prior knowledge for probe design and an inability to allow scanning of poorly methylated and repetitive sequence regions^18^. Although MeDIP-Seq has less genomic coverage and limited resolution (about 200 bp) in comparison with that of BS-seq^19^,^20^, it is a suitable and cost-effective approach for comparative analyses of animal methylomes using small amounts of DNA, because it uses immunoprecipitation with an antibody against 5-methylcytosine to enrich methylated DNA fragments and enables rapid identification of multiple universal CpG sites^19^. This method has been widely used to analyze the genome-wide methylation profiles of many animals, including chickens^21^,^22^, rats^23^, honeybees^24^, silkworms^25^, horses^26^, and cattle^27^.

With the development of high-throughput sequencing technologies, comprehensive analysis of the mammalian genome has altered our view of the genetic basis of phenotypes. In previous studies on the molecular mechanism of phenotype difference between breeds, most of them on pigs focused on mRNA and miRNA transcriptomes^28^,^29^,^30^,^31^. DNA methylation of pigs has been intensively affected by artificial selection during domestication and breeding. Recently, several DNA methylome studies in healthy pigs have been reported. Yang *et al*. assessed the extent and pattern of cytosine methylation in six tissues from the Laiwu swine strain using the fluorescence-labeled methylation-sensitive amplified polymorphism method^32^, but this method was limited by the sequence context of the chosen enzyme. Li *et al*. reported the first comprehensive methylome map of adipose and skeletal muscle tissues of pigs with different phenotypes and investigated the relationship between DNA methylation and fat deposition using MeDIP-seq^33^,^34^. The genome-wide DNA methylation profiles of Berkshire, Duroc, and Landrace pigs show that these breeds exhibit both conserved and divergent DNA methylation patterns across their genomes^35^. Moreover, RRBS studies of the pig genome have been conducted using different types of tissue^36^,^37^, whereas aging skeletal muscle^38^ has been assessed using MeDIP-seq; these results indicate that pigs are an ideal model organism for biomedical studies related to aging. Another RRBS analysis reported differences in prenatal and postnatal DNA methylation in intestinal tissue^39^.

In this study, we used MeDIP-seq to carry out genome-wide DNA methylation analysis of blood leukocytes from three pig breeds with substantial phenotype differences in body size, growth rate, and fat content: Tongcheng, Landrace, and Wuzhishan. We obtained comprehensive DNA methylation profiles for these pig breeds and identified differentially methylated genes (DMGs) related to development and metabolism that might contribute to phenotypic variance among pig breeds.

## Results

### Mapping and statistical analysis of MeDIP-seq reads

**Figure 1:**
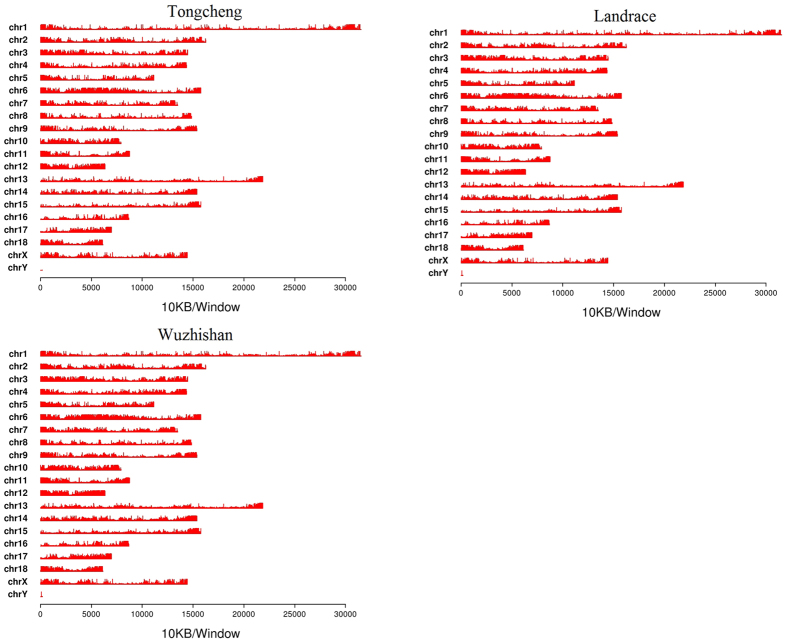
Chromosome distribution of reads in Tongcheng (**A**), Landrace (**B**), and Wuzhishan (**C**) pigs. The distribution of reads in chromosomes 1–18 and the X chromosome of the pig genome are shown in red for each sample. MeDIP-seq reads were plotted in 10-kb windows along the chromosome.

Blood leukocyte DNA from 10 individuals per breed was used to generate one pooled sample for the Tongcheng, Landrace, and Wuzhishan pig breeds. Next, we carried out genome-wide DNA methylation profiling using MeDIP-seq. After removing reads that were contaminated, of low quantity, or only contained adaptor reads, approximately 68 million paired-end raw reads were obtained for each library. In Tongcheng, Landrace, and Wuzhishan pigs, 78.29%, 83.57%, and 82.36% of the reads, respectively, were mapped to *Sus scrofa* genome assembly 10.2 (Table 1); the uniquely mapped reads covered 59.43%, 67.92%, and 65.80%, respectively, of the pig reference genome. MeDIP-seq reads were detected in all chromosomal regions (SSC1-18 and the X chromosome; Fig. 1).

**Figure 2:**
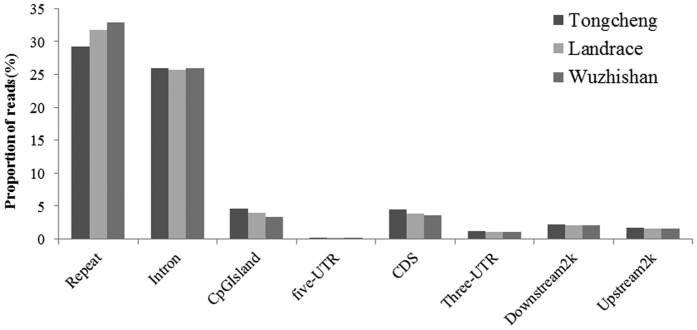
Read distribution in different elements of the Tongcheng, Landrace, and Wuzhishan pig genomes. The y-axis is the proportion of reads. The x-axis shows the different components of the genome.

**Figure 3:**
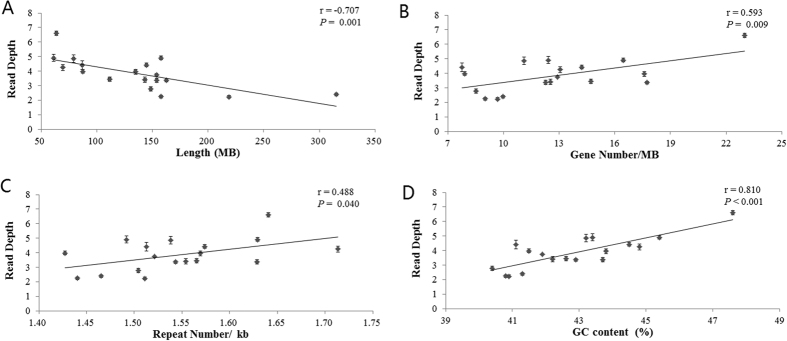
DNA methylation level with genomic features. (**A**) Pearson’s correlation between DNA methylation level and autosome length in pigs (chromosomes 1–18). (**B**) Pearson’s correlation between DNA methylation level and gene density of pig autosomes. (**C**) Pearson’s correlation between DNA methylation level and the repeat density of pig autosomes. (**D**) Pearson’s correlation between DNA methylation level and the GC content of pig autosomes.

Analysis of read distributions in different components of the *Sus scrofa* genome showed that the uniquely mapped reads were mainly present in repeat elements and intron regions. The proportion of unique MeDIP-seq reads mapped to repeat elements was 29.21%, 31.78%, and 32.87% in Tongcheng, Landrace, and Wuzhishan pigs, respectively (Fig. 2). Reads in the repeat elements were primarily concentrated in SINE/tRNA-Glu and LINE/L1 elements and accounted for 79% of the total repeat element reads (Table 2). Approximately 25% of unique MeDIP-seq reads were found in intron regions. The proportion of reads uniquely mapped to CpG islands (CpGIs) was only 4.59%, 4.00%, and 3.30% in Tongcheng, Landrace, and Wuzhishan pigs, respectively (Fig. 2). We also observed that methylation levels were negatively correlated with chromosome length (Pearson’s r = −0.707, *p* = 0.001) and positively correlated with repeat density (Pearson’s r = 0.488, *p* = 0.040), gene density (Pearson’s r = 0.593, *p* = 0.009), and GC percentage (Pearson’s r = 0.810, *p* = 4.59 × 10^−5^) (Fig. 3).

### Validation of MeDIP-seq data via quantitative MassARRAY methylation analysis

In order to confirm the reliability of the MeDIP-seq results, three regions showing high methylation and one region showing low methylation were randomly selected for validation using Sequenom MassArray methylation analysis, by which the methylated or unmethylated DNA fragments were measured quantitatively by mass spectrometry analysis. The bisulfite sequencing results were in accordance with our MeDIP-seq data (Figure S5).

### Genome-wide DNA methylation patterns of pigs

**Figure 4:**
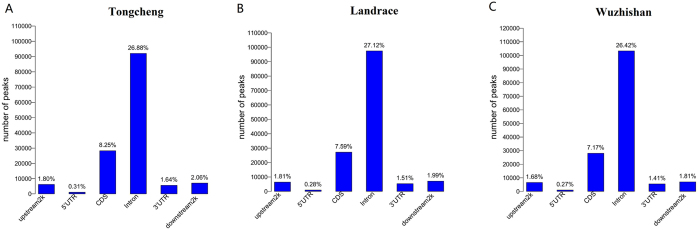
Peak distribution in different components of the genome. The y-axis is the number of peaks. The x-axis shows the different components of the genome. (**A**) Tongcheng; (**B**) Landrace; (**C**) Wuzhishan.

**Figure 5:**
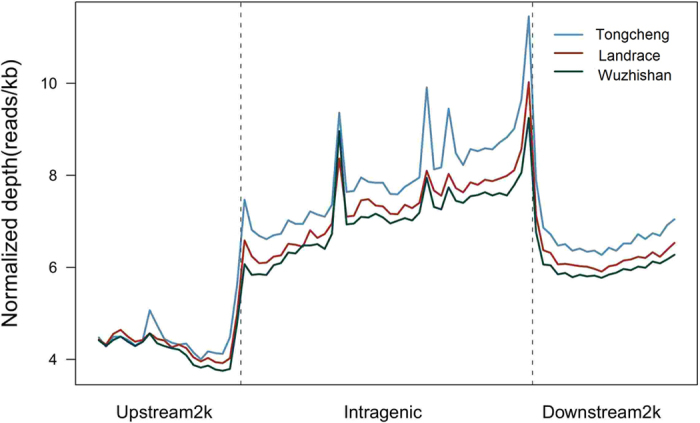
DNA methylation distribution in pigs. The DNA methylation profile for each gene region is shown by the reads that aligned with the unique locus in the genome. The gene region was defined as the regions that contained a 2-kb region upstream of the TSS, the gene body from TSS to TTS, and a 2-kb region downstream of the TTS. The 2-kb upstream and downstream regions were split into 20 non-overlapping windows, and the average alignment depth was calculated for each window. In the gene body, each gene was split into 40 equal windows, and the average alignment depth was calculated for each window. The y-axis is the average of the normalized depth for each window.

To decipher the genome-wide DNA methylation profiles of different pig breeds, the uniquely mapped reads were used to detect methylated peaks. We detected 342,383, 359,251, and 390,799 methylated peaks in Tongcheng, Landrace, and Wuzhishan pigs, respectively (Table S1,S2,S3). The mean length of the peaks was approximately 300 bp. The methylated peaks identified in Tongcheng, Landrace, and Wuzhishan pigs covered 4.38%, 5.21%, and 5.76%, respectively, of the pig reference genome (Table 3). These results indicate that only a small fraction of the pig genome was methylated. There were fewer than 20 CpGs in most of the peaks; the number of CpGs in the peaks was shown in Figures S1–S3. Analysis of the peak distribution showed that the majority of peaks were present in intergenic regions, followed by intronic and exonic regions (Fig. 4). Analysis of peak coverage showed that the genome coverage was approximately 21%, 58.8%, 75.7%, 10%, 28.8%, and 23.3%, in the 2-kb upstream, 5′-UTR, exonic, intronic, 3′-UTR, and 2-kb downstream regions, respectively (Figure S4). We also analyzed the distribution of DNA methylation in 2-kb regions upstream of transcription start sites (TSS), in gene bodies, and in the 2-kb region downstream of transcription termination sites (TTS). Generally, gene body regions showed levels of DNA methylation higher than those of the 5′ and 3′ flanking regions of genes. In the gene body region, Tongcheng pigs had the highest methylation level, whereas Wuzhishan pigs had the lowest methylation level (Fig. 5).

### Distribution of DNA methylation in CpGIs

CpGIs that overlapped with the methylation peaks were considered methylated CpGIs. Of all of the CpGIs in the pig genome, 6,582 (15.08%), 6,369 (14.59%), and 5,516 (12.64%) were methylated in Tongcheng, Landrace, and Wuzhishan pigs, respectively (Table S4,S5,S6). Most CpGIs in each genome were unmethylated; however, CpGIs were least likely to be methylated in Wuzhishan pigs and most likely to be methylated in Tongcheng pigs.

### Gene ontology analysis of methylated genes

Genes that overlapped with the methylation peaks in the promoters or gene body regions were considered as methylated genes. We identified 17,188, 17,359, and 17,545 methylated genes in Tongcheng, Landrace, and Wuzhishan pigs, respectively (Table S7,S8,S9). Interestingly, seven methylated miRNAs were identified in our study: ssc-mir-935, ssc-mir-7144, ssc-mir-671, ssc-mir-451, ssc-mir-21, ssc-mir-1306, and ssc-mir-127.

**Figure 6:**
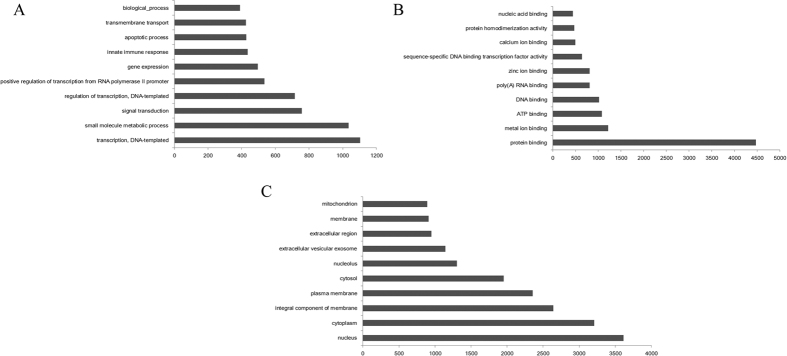
GO categories of methylated genes. (**A**) biological process; (**B**) molecular function; (**C**) cellular component.

Gene ontology (GO) analysis was performed for the methylated genes detected in all three pig breeds. A total of 11,956 methylated genes were annotated in three categories: biological process, cellular component, and molecular function. The methylated genes were mainly enriched in the following biological process terms: transcription, DNA-templated (1,103; 6.10%); small molecule metabolic process (1,036; 5.72%); signal transduction (756; 4.18%), regulation of transcription, DNA-templated (716; 3.96%), and positive regulation of transcription from the RNA polymerase II promoter (536; 2.96%) (Fig. 6A). The methylated genes were enriched in the following cellular component terms: nucleus (3,615; 19.99%), cytoplasm (3,206; 17.73%), integral component of membrane (2,641; 14.60%), plasma membrane (2,354; 13.01%), and cytosol (1,952; 10.79%) (Fig. 6B). The methylated genes were enriched in the following molecular function terms: protein binding (4,470; 24.72%), metal ion binding (1,216; 6.72%), ATP binding (1082; 5.98%), DNA binding (1,020, 5.64%), and poly(A) RNA binding (808; 4.47%) (Fig. 6C).

### Differentially methylated genes

**Figure 7:**
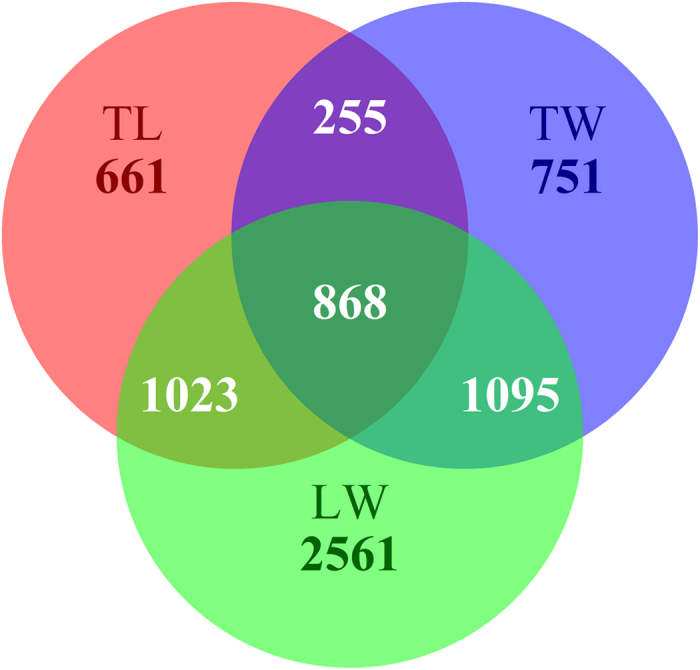
Unique or shared DMGs among three contrasts. TL: Tongcheng Vs. Landrace pigs. TW: Tongcheng Vs. Wuzhishan pigs. LW: Landrace Vs. Wuzhishan pigs.

Differentially methylated genes (DMGs) were identified with a change of more than four-fold in coverage and FDR < 0.001. We detected 2,807 DMGs between Landrace and Tongcheng pigs (Table S10), 2,969 DMGs between Tongcheng and Wuzhishan pigs (Table S11), and 5,547 DMGs between Landrace and Wuzhishan pigs (Table S12). In a recent study, we identified more differentially expressed genes in the comparison of skeletal muscle from Landrace and Wuzhishan pigs than were identified in comparisons of Landrace vs. Tongcheng pigs and Tongcheng vs. Wuzhishan pigs^28^, indicating that the difference between Landrace and Wuzhishan pigs is larger than that between Tongcheng and Landrace or Wuzhishan pigs. Moreover, 868 DMGs were observed in all three comparisons (Fig. 7).

### Gene ontology analysis of differentially methylated genes

**Figure 8:**
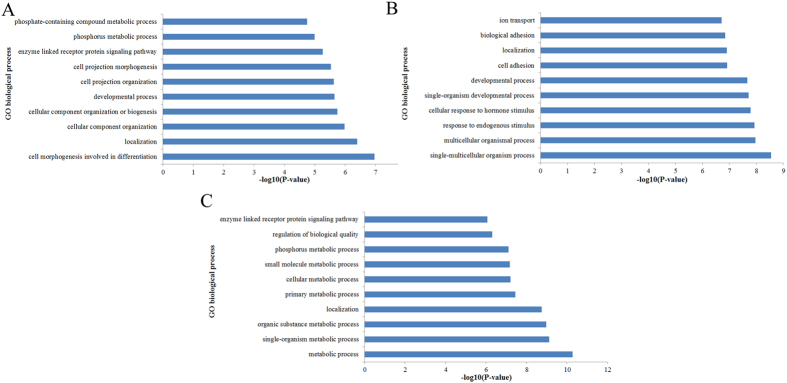
The top 10 GO terms significantly enriched for differentially methylated genes in the three contrasts. (**A**) Tongcheng Vs. Landrace pigs, (**B**) Tongcheng Vs. Wuzhishan pigs, (**C**) Landrace Vs. Wuzhishan pigs. GO analysis was conducted by Bioconductor package GOstats^82^.

GO enrichment analysis was performed to gain insight into the biological processes in which the DMGs might be involved. In the Tongcheng vs. Landrace group, the most significantly enriched terms were cell morphogenesis involved in differentiation, localization, cellular component organization, developmental process, cell projection organization, and cell projection morphogenesis (Fig. 8A). In the Tongcheng vs. Wuzhishan group, the most significantly enriched terms were single-multicellular organism process, multicellular organismal process, response to endogenous stimulus, cellular response to hormone stimulus, single-organism developmental process, developmental process, cell adhesion, and localization (Fig. 8B). In the Landrace vs. Wuzhishan group, the most significantly enriched terms were metabolic process, single-organism metabolic process, organic substance metabolic process, localization, primary metabolic process, cellular metabolic process, small molecule metabolic process, and phosphorus metabolic process (Fig. 8C).

### KEGG pathway enrichment analysis of differentially methylated genes

**Figure 9:**
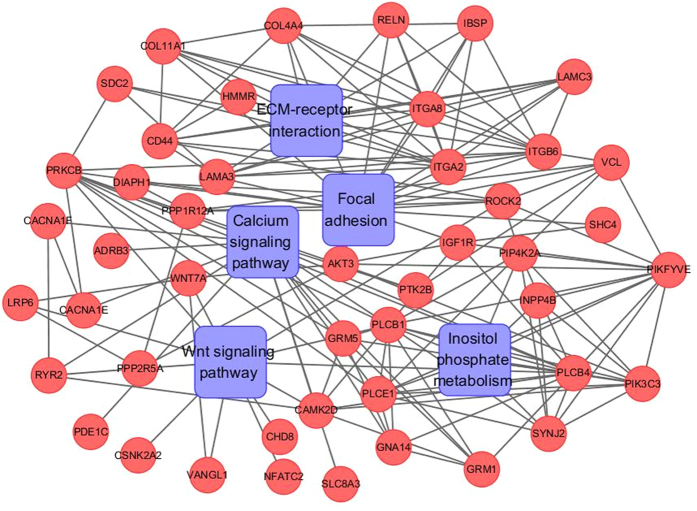
KEGG enrichment pathways and protein-protein interaction network analysis of differentially methylated genes present in all three contrasts (Tongcheng Vs. Landrace, Tongcheng Vs. Wuzhishan, and Landrace Vs. Wuzhishan pigs).

A KEGG pathway analysis was performed to investigate the pathways in which the DMGs might be involved. The Tongcheng vs. Landrace DMGs were significantly enriched in the Wnt signaling pathway, metabolic pathways, and the phosphatidylinositol signaling system. The Tongcheng vs. Wuzhishan DMGs were significantly enriched in the PPAR signaling pathway, fatty acid metabolism, the MAPK signaling pathway, the phosphatidylinositol signaling system, ECM-receptor interaction, the calcium signaling pathway, and biosynthesis of unsaturated fatty acids. The Landrace vs. Wuzhishan DMGs were significantly enriched in metabolic pathways, the phosphatidylinositol signaling system, the PPAR signaling pathway, the Wnt signaling pathway, salivary secretion, and glycerophospholipid metabolism. The shared DMGs identified in all three comparisons were significantly enriched in pathways related to development and metabolism, including the Wnt signaling pathway, the calcium signaling pathway, inositol phosphate metabolism, ECM-receptor interaction, and focal adhesion. There were 46 differentially methylated genes in these five pathways. The protein-protein interaction network analysis indicated that these DMGs were highly correlated with each other (Fig. 9).

### Candidate DMGs associated with phenotype differences

Many transcriptome and association studies have explored the molecular mechanisms underlying phenotypic variance in pigs, providing a foundation for our investigation of the involvement of DMGs in phenotypic variance. Candidate DMGs were identified according to the following criteria: (1) genes were differentially methylated in all the three comparisons; (2) genes were enriched in pathways related to development and metabolism; (3) genes were differentially expressed between pigs with different phenotypes or associated with economic traits reported by previous studies. The application of these criteria led to the identification of 32 candidate DMGs associated with phenotype differences in different pig breeds, including some well-studied genes such as *ADRB3, IGFR1, ITGA2, ITGA8, CAMK2D,* and *ROCK2* (Table S13).

## Discussion

Recently, several methylome studies using MeDIP-seq or RRBS in healthy pigs have been reported^33^,^34^,^35^,^36^,^37^,^38^,^39^. However, there are no reports of methylome analysis of blood leukocytes in pigs with phenotypic divergence. Blood is the most common source of biomarkers and materials for genetic studies, because it is easily collected and interacts with all organs. Global analysis of methylation profiles using blood leukocyte DNA has been widely used to explain phenotypic differences in growth, metabolism, body size, and obesity in humans and other animals^40^,^41^,^42^,^43^. Moreover, transcriptome analysis in pigs demonstrates the usefulness of blood in elucidating biological processes associated with various traits, such as feed efficiency^44^,^45^ and immunity^46^,^47^. Our study first systematically compared the genome-wide methylation profiles of blood leukocytes from three pig breeds with different phenotypes (Tongcheng, Landrace, and Wuzhishan pigs) and identified DMGs that might affect the development of different phenotypes during artificial selection.

Our results indicate that the analyzed pigs had DNA methylation patterns similar to those of other mammals^26^,^27^. Read distribution analysis found that uniquely mapped reads were enriched in repeat elements and intron regions, which was consistent with previous findings regarding hypermethylated repetitive sequences in other species^23^,^27^,^48^. We also found that methylation levels were negatively correlated with chromosome length and positively correlated with repeat density, gene density, and GC percent, in accordance with previous MeDIP-seq studies using pigs^33^,^38^. Using RRBS in five tissue types from pigs, Choi *et al*. also found that the level of CpG methylation of each chromosome was positively correlated with the GC percentage of the chromosomes, but they did not observe any correlation between gene number and CpG methylation of chromosomes^36^, perhaps because of the greater genomic coverage of MeDIP-seq in comparison with that of RRBS.

Previous studies showed that repeat elements account for approximately 40% of the pig genome. LINE/L1 and SINE/tRNA-Glu are the most abundant class of repeats in pigs, accounting for 68% of total repeat elements and 27.4% of the pig genome^3^,^49^,^50^. Our results indicate that LINE/L1 and SINE/tRNA-Glu are the predominant methylated DNA repeats in the analyzed pig genomes.

We found that only a small fraction of the pig genome was methylated (methylated peak regions covered approximately 5% of the genome in each sample), similar to the results of a study on the bovine placental genome^27^. Approximately 15% of CpGIs in each pig breed were methylated, which indicated that most of the CpGIs remained hypomethylated. We also found that gene body regions show a level of DNA methylation much higher than those of 5′ and 3′ flanking regions. Hypermethylation of the gene body regions in the pig genome further indicated that this was probably a mechanism of gene expression regulation that was conserved among species. Most of the genes had one or more methylated regions in their promoter and gene body regions. This phenomenon could be explained by the high proportion of methylated CpG sites in mammals^51^ and the high sequencing depth of our data.

GO biological process analysis was performed to investigate the potential functions of DMGs responsible for phenotype differences among different pig breeds. We found that DMGs involved in developmental processes and metabolic processes were significantly enriched in all three comparisons. Developmental process and fatty acid metabolic process were the major biological process terms enriched in the set of differentially expressed genes (DEGs) identified in an analysis of two phenotypically extreme pigs^52^. A transcriptome analysis of three types of tissue from a full-sib pair with extreme phenotypes in growth and fat deposition also showed that metabolic process was one of the most enriched terms in the set of DEGs^53^. The differences in fat content, body size, and muscle growth rate among Tongcheng, Landrace, and Wuzhishan pigs might be mediated by methylation modification. Compared with Landrace pigs, Wuzhishan pigs are smaller and not as tall. We found that 6 DMGs (*BBS7, EFEMP1, EIF2AK3, FBN1, FBN2, HHIP*) between these two breeds, which are involved in developmental processes, are associated with human height and stature^54^. For example, *FBN1* encodes a fibrillin family protein associated with Marfan syndrome and contains large-effect mutations for height that are explained by allelic heterogeneity^55^. The *EFEMP1* gene has been shown to affect human height in 6 genome-wide association studies and a confirmation study in cattle^54^,^56^. Genes related to fatty acid metabolism and oxidation, including *ACADM, PRKAA1, ACACB, CAB39L, CPT2*, and *ACSL1*, were overrepresented in the sets of DMGs identified among the three breeds. It is worth mentioning that the *ACACB* gene, a key regulator of fatty acid oxidation, is differentially expressed between Basque pigs (obese-type) and Large White pigs (lean-type)^57^. A recent study also showed that *ACACB* was expressed much more highly in Wuzhishan pigs in comparison with Landrace and Tongcheng pigs^28^. Li *et al*. reported that *CPT2, ACACB, ACADM*, and *ACSL1* genes were differentially expressed between Wannanhua (obese-type) and Large White pigs^58^. These results suggest that these pigs have a distinctive developmental process and metabolic capacity and indicated that differences in DNA methylation might underlie differences in development and metabolism among pig breeds.

For the DMGs identified in all three comparisons (Tongcheng vs. Landrace, Tongcheng vs. Wuzhishan, and Landrace vs. Wuzhishan), pathway enrichment analysis showed significant enrichment in several important pathways related to developmental and metabolic processes, including the Wnt signaling pathway, the calcium signaling pathway, inositol phosphate metabolism, focal adhesion, and ECM-receptor interaction. The Wnt signaling participates in multiple developmental events during embryogenesis^59^ and is involved in satellite cell proliferation and differentiation during adult skeletal muscle regeneration^60^. Wnt signaling is also essential for muscle fiber growth and maintenance because it regulates slow and fast twitch muscle myofibrillogenesis^61^. The calcium signaling participate in many processes during animal embryonic development^62^ and plays a crucial role in muscle function and plasticity^63^. Inositol phosphate regulates glycolytic and lipid metabolism and functions in cell signaling and cell growth^64^,^65^. The extracellular matrix has an important role in tissue morphogenesis and adipogenesis^66^ and is involved in the regulation of skeletal muscle development^67^. Therefore, these pathways might regulate development and metabolism, and significantly contribute to phenotype variance of different breeds in pigs.

DNA methylation aberration in gene promoters and gene bodies influences gene expression levels^68^. SNP variations are also associated with differences in methylation and gene expression levels^69^. To identify candidate DMGs associated with phenotype differences in different pig breeds, we integrated analysis of DMGs with previous transcriptome and association studies, leading to the identification of 32 candidate DMGs, most of which were differentially expressed among different pig breeds or during development (Table S13). For example, *COL11A1*, a major ECM component, was expressed at lower levels in Korean native pigs than in Yorkshire pigs, indicating a difference in ECM structure between the breeds^70^. *CAMK2D* was specifically expressed in pig skeletal muscle and regulated by miR-1207-5p^71^; it was expressed at a much lower level in Wuzhishan pigs than in Landrace and Tongcheng pigs^28^. The *ADRB3* gene, a major mediator of lipolytic and thermogenic effects in adipose tissue, was reported to associate with fatness traits by several studies^72^,^73^,^74^. *IGF1R* was identified as a potential candidate gene\for postnatal growth and carcass composition traits in pigs^75^ and plays a crucial role in skeletal muscle development and differentiation^76^. *ROCK2* plays a key role in the control of skeletal and cardiac myocyte cell differentiation and is differentially expressed during skeletal muscle development^77^. We hypothesize that these DMGs might contribute to phenotype differences in Tongcheng, Landrace, and Wuzhishan pigs, but the specific effects of methylation on expression of these genes during development and metabolism requires further study.

This study provides comprehensive DNA methylation profiles of whole blood from Tongcheng, Landrace, and Wuzhishan pigs. These DNA methylation profiles provide new clues for deciphering epigenetic regulation mechanisms in mammals and identified novel candidate genes associated with phenotype differences among pig breeds.

## Materials and Methods

### Animals

Ten female adult pigs (240 days after birth) of each breed (Tongcheng, Landrace, and Wuzhishan) were utilized for DNA methylation analysis. Unrelated individuals of each breed were chosen based on their pedigrees. All pigs used in our study were raised under the same feeding and management practices at our experimental farm in Beijing. Whole blood was collected from each pig via the precava according to the animal procedures defined by national and local animal welfare bodies. The collected blood samples were stored at −20 °C. All animal procedures were conducted according to protocols approved by Hubei Province, P.R. China, for the Biological Studies Animal Care and Use Committee.

### DNA extraction and preparation for MeDIP-seq

Genomic DNA from blood leukocytes was isolated by phenol-chloroform extraction. DNA quality and concentration were evaluated by agarose gel electrophoresis and spectrophotometry. For each breed, blood leukocyte DNA from 10 pigs was mixed in equal amounts to generate a pooled sample using the Quant-iT dsDNA HS Assay Kit (Invitrogen, Carlsbad, CA, USA). Subsequently, genomic DNA was fragmented using a Covarias sonication system to produce 100–500-bp DNA fragments. After end repair, base addition at the 3′-end and adaptor ligation were performed using Illumina’s Pair-End DNA Sample Prep Kit following the manufacturer’s instructions (Illumina, San Diego, CA, USA). Double-stranded DNA was denatured to single-stranded DNA and immunoprecipitated using anti-5-methylcytosine mouse monoclonal antibodies (anti-5mc) (Calbiochem, San Diego, CA, USA). MeDIP products were validated by real-time quantitative PCR (qPCR) using SYBR Green Master Mix (Invitrogen, Carlsbad, CA, USA) and primers for positive and negative control regions supplied in the MeDIP kit (Diagenode, Sparta, NJ, USA). qPCR validation procedures consisted of 95 °C for 5 min, followed by 40 cycles 95 °C for 15 s, and 60 °C for 1 min.

MeDIP DNA was purified with a ZYMO DNA Clean & Concentrator-5 column following the manufacturer’s instructions and amplified by adaptor-mediated PCR (Zymo Research, Orange, CA, USA). After excising amplified DNA between 220 and 320 bp in length on a 2% agarose gel, amplification quality and quantity were evaluated using an Agilent 2100 Analyzer and the DNA 1000 Nano Chip Kit (Agilent Technologies, Santa Clara, CA, USA). Qualified libraries were subjected to high-throughput sequencing using an Illumina Genome Analyzer II to generate 49-bp paired-end reads for methylation profile analysis by the Beijing Genomics Institute (BGI, Shenzhen, Guangdong, China).

### Data Analysis

After obtaining the raw data from Illumina sequencing, reads containing adapters, unknown, or low quality bases were filtered out. The remaining clean reads were then aligned to *Sus scrofa* reference genome build 10.2^3^ by SOAPaligner v2.21 (http://soap.genomics.org.cn/)^78^; mismatches no larger than 2-bp were allowed, and the uniquely mapped reads were retained for further analysis.

Annotation information of the porcine reference genome was downloaded from the Ensembl public FTP site (ftp://ftp.ensembl.org/pub/release-78/gtf/sus_scrofa/Sus_scrofa.Sscrofa10.2.78.gtf.gz). The region from the TSS to TTS was defined as the gene body region, and the genomic region 2-kb upstream of the TSS was considered as the proximal promoter region. CpGI and repeat annotation information was downloaded from the UCSC public FTP site (http://hgdownload.cse.ucsc.edu/goldenPath/susScr3/database/), and analyses of read distributions over repeats were carried out by RepeatMasker version open-4.0.3 (http://www.repeatmasker.org/)^79^. Model-based Analysis of ChIP-Seq (MACS v1.4.2) (http://liulab.dfci.harvard.edu/MACS/) was used to scan the methylated peaks in the porcine genome with default parameters^80^. GO analysis of the methylated genes was performed. GO term information was obtained from the UniProtKB-GOA database (http://www.ebi.ac.uk/GOA/).

Differentially methylated regions were identified by Bioconductor package edgeR using the exact test for negative binomial distribution^81^. Genes that exhibited a difference of more than four-fold in the number of reads between different samples and FDR <0.001 (adjusted by the Benjamini-Hochberg method) were identified as differentially methylated genes (DMGs). Gene ontology and KEGG pathway enrichment analyses of the DMGs were performed using Bioconductor package GOstats^82^. DMGs involved in KEGG pathways related to development and metabolism were submitted to STRING v9.1 for protein-protein interaction (PPI) network analyses (http://string-db.org/)^83^. Visualization of the PPI network was performed using Cytoscape version 3.2.1^84^.

### Sequenom MassARRAY quantitative methylation analysis

For MeDIP-Seq validation, genomic DNA isolated from blood leukocytes from three additional unrelated pigs (female, 8 months old) of each breed was treated with sodium bisulfite using the EZ DNA Methylation-Gold Kit (Zymo Research, Irvine, CA, USA) according to the manufacturer’s instructions. Specific primers of selected regions were designed using Epidesigner software (http://www.epidesigner.com/). Quantitative methylation analysis was performed on the Sequenom MassARRAY platform by Bio Miao Biological Technology (Beijing, China). The quantitative methylation data for each CpG site or multiple CpG sites were analyzed with EpiTYPER software v1.0 (Sequenom).

### Online data deposition

The MeDIP-seq raw data from this study have been deposited in NCBI Sequence Read Archive with accession number SRP062813. (http://www.ncbi.nlm.nih.gov/Traces/sra/).

## Additional Information

**How to cite this article**: Yang, Y. *et al*. Genome-wide analysis of DNA methylation in obese, lean, and miniature pig breeds. *Sci. Rep.* **6**, 30160; doi: 10.1038/srep30160 (2016).

## Supplementary Material

Supplementary Figure S1-S5

Supplementary Table S1

Supplementary Table S2

Supplementary Table S3

Supplementary Table S4

Supplementary Table S5

Supplementary Table S6

Supplementary Table S7

Supplementary Table S8

Supplementary Table S9

Supplementary Table S10

Supplementary Table S11

Supplementary Table S12

Supplementary Table S13

## Figures and Tables

**Table 1:** Mapping results of MeDIP-seq data.

	Tongcheng	Landrace	Wuzhishan
Insert Size (bp)	320	242	353
Read Length (bp)	49	49	49
Total raw reads	68,181,818 (3G)	68,181,818 (3G)	68,181,818 (3G)
Total Mapped Reads	53,377,686	56,976,504	56,152,638
Percentage of mapped reads in total reads (%)	78.29	83.57	82.36
Total Mapped Bases (bp)	2,615,506,614	2,791,848,696	2,751,479,262
Total Unique Mapped Reads	34,062,656	38,929,967	37,714,905
Total Unique Mapped Bases (bp)	1,669,070,144	1,907,568,383	1,848,030,345
Percentage of unique mapped reads (%)	49.96	57.10	55.32

**Table 2:** Distribution of reads in repeat elements.

Repeat type	Tongcheng (%)	Landrace (%)	Wuzhishan (%)
SINE/tRNA-Glu	6573616(60.02)	8458026(61.68)	8609552(62.56)
LINE/L1	2099477(19.17)	2555829(18.64)	2497220(18.14)
LINE/L2	351925(3.21)	456016(3.33)	458375(3.33)
LTR/ERV1	351849(3.21)	419673(3.06)	409342(2.97)
LTR/ERVL-MaLR	338203(3.09)	393497(2.87)	400515(2.91)
SINE/MIR	277874(2.54)	326031(2.38)	331960(2.41)
LTR/ERVL	245616(2.24)	282058(2.06)	285775(2.08)
DNA/hAT-Charlie	190622(1.74)	218446(1.59)	218648(1.59)
DNA/TcMar-Tigger	108035(0.99)	124841(0.91)	124661(0.91)
DNA/hAT-Tip100	43569(0.4)	47746(0.35)	48882(0.36)
LTR/ERVK	37358(0.34)	46833(0.34)	42005(0.31)
DNA/Sola	34916(0.32)	42433(0.31)	36540(0.27)
DNA/Novosib	34571(0.32)	41400(0.3)	35622(0.26)
LINE/Penelope	38924(0.36)	35396(0.26)	27769(0.2)
DNA/CMC-EnSpm	29031(0.27)	34847(0.25)	29227(0.21)
others	196192(1.76)	228737(1.62)	206892(1.45)

**Table 3:** Statistic results of Peak.

Sample	Total Peaks	Peak Mean Length (bp)	Peak Median Length (bp)	Peak Total Length (bp)	Peak Covered Size In Genome (%)
**Tongcheng**	342,383	359.46	300	123,072,236	4.38
**Landrace**	359,251	407.28	343	146,314,131	5.21
**Wuzhishan**	390,799	413.88	344	161,744,778	5.76
