# Supplementary material for: Genome-wide analysis of DNA methylation in obese, lean, and miniature pig breeds
Source: Sci Rep. 2016 Jul 22;6:30160. doi: 10.1038/srep30160 (PMC4957084; doi:10.1038/srep30160)
Supplement: Supplementary Figure S1-S5 [file srep30160-s1.doc]

**Supplementary Information for**

**Genome-wide analysis of DNA methylation in obese, lean, and miniature pig breeds**

Yalan Yang^1,2†^, Rong Zhou^1†^, Yulian Mu^1^, Xinhua Hou^1^, Zhonglin Tang^1,2*^, Kui Li^1,2^

1. State Key Laboratory of Animal Nutrition, Institute of Animal Science, Chinese Academy of Agricultural Sciences, Beijing 100193, China.;
2. Agricultural Genome Institute at Shenzhen, Chinese Academy of Agricultural Sciences, Shenzhen, 518124, China.

^†^ These authors contributed equally to this work

^*^Corresponding author: Zhonglin Tang, State Key Laboratory of Animal Nutrition, Institute of Animal Science, Chinese Academy of Agricultural Sciences, Email: tangzhonglin@caas.cn.

**Supporting information**

**Figure S1.** Number of CpGs in methylated peaks of Tongcheng pigs.

**Figure S2.** Number of CpGs in methylated peaks of Landrace pigs.

**Figure S3.** Number of CpGs in methylated peaks of Wuzhishan pigs.

**Figure S4.** Peak genome coverage in different elements of the genome. (A) Tongcheng; (B) Landrace; (C) Wuzhishan. Genome coverage was calculated as follows: (base number of peaks in component / total base number of the component) × 100

**Figure S5**. Validation of MeDIP-Seq data by quantitative MassARRAY methylation analysis in four selected regions.

**Table S1.** Methylated peaks in Tongcheng pigs.

**Table S2.** Methylated peaks in Landrace pigs.

**Table S3.** Methylated peaks in Wuzhishan pigs.

**Table S4.** Methylated CpGIs in Tongcheng pigs.

**Table S5.** Methylated CpGIs in Landrace pigs.

**Table S6.** Methylated CpGIs in Wuzhishan pigs.

**Table S7.** Methylated genes in Tongcheng pigs.

**Table S8.** Methylated genes in Lndrace pigs.

**Table S9.** Methylated genes in Wuzhishan pigs.

**Table S10.** Differentially methylated genes between Tongcheng and Landrace pigs.

**Table S11.** Differentially methylated genes between Tongcheng and Wuzhishan pigs.

**Table S12.** Differentially methylated genes between Landrace and Wuzhishan pigs.

**Table S13** Differentially methylated genes potentially related to pig development and metabolism.


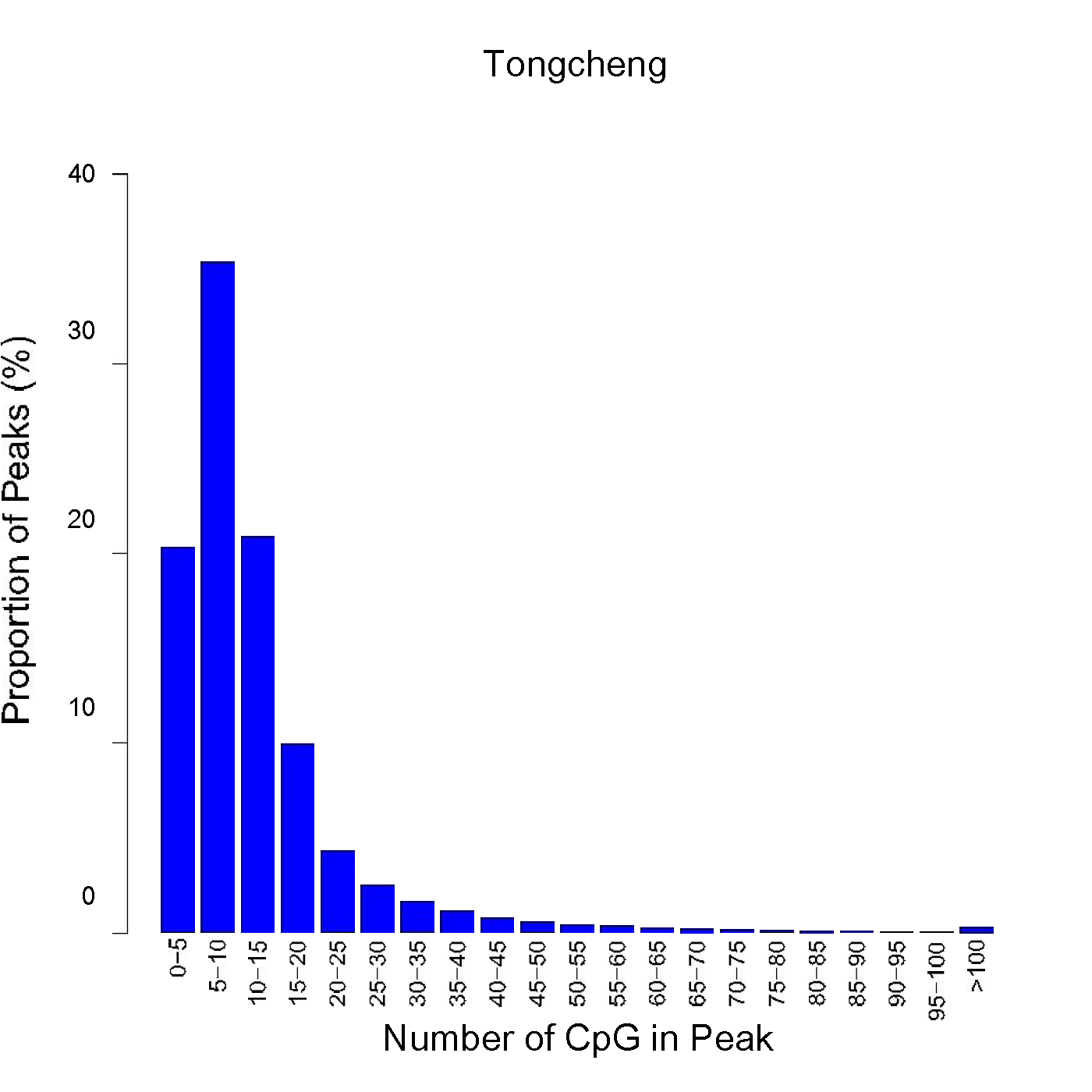


**Figure S1.** Number of CpGs in methylated peaks of Tongcheng pigs.


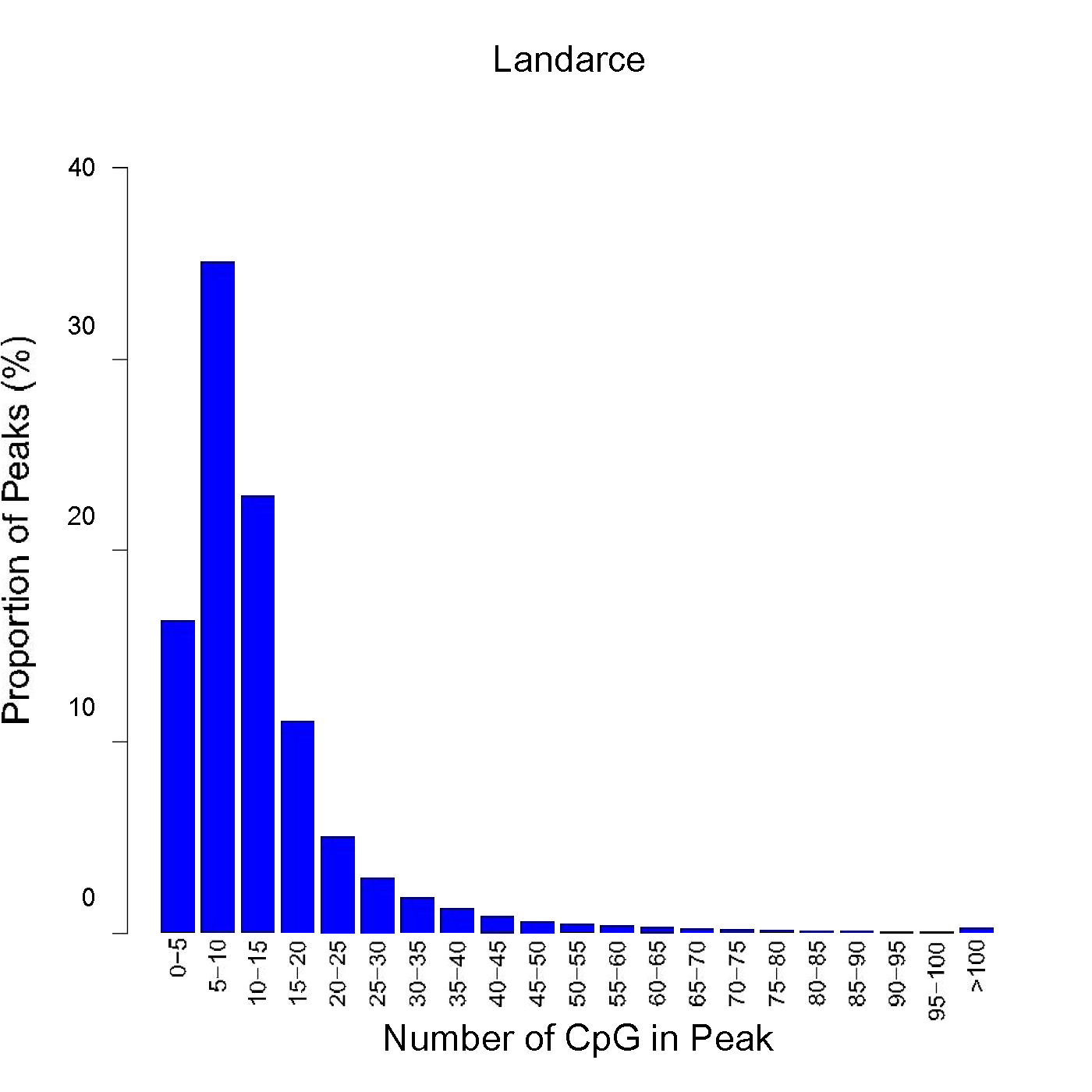


**Figure S2.** Number of CpGs in methylated peaks of Landrace pigs.


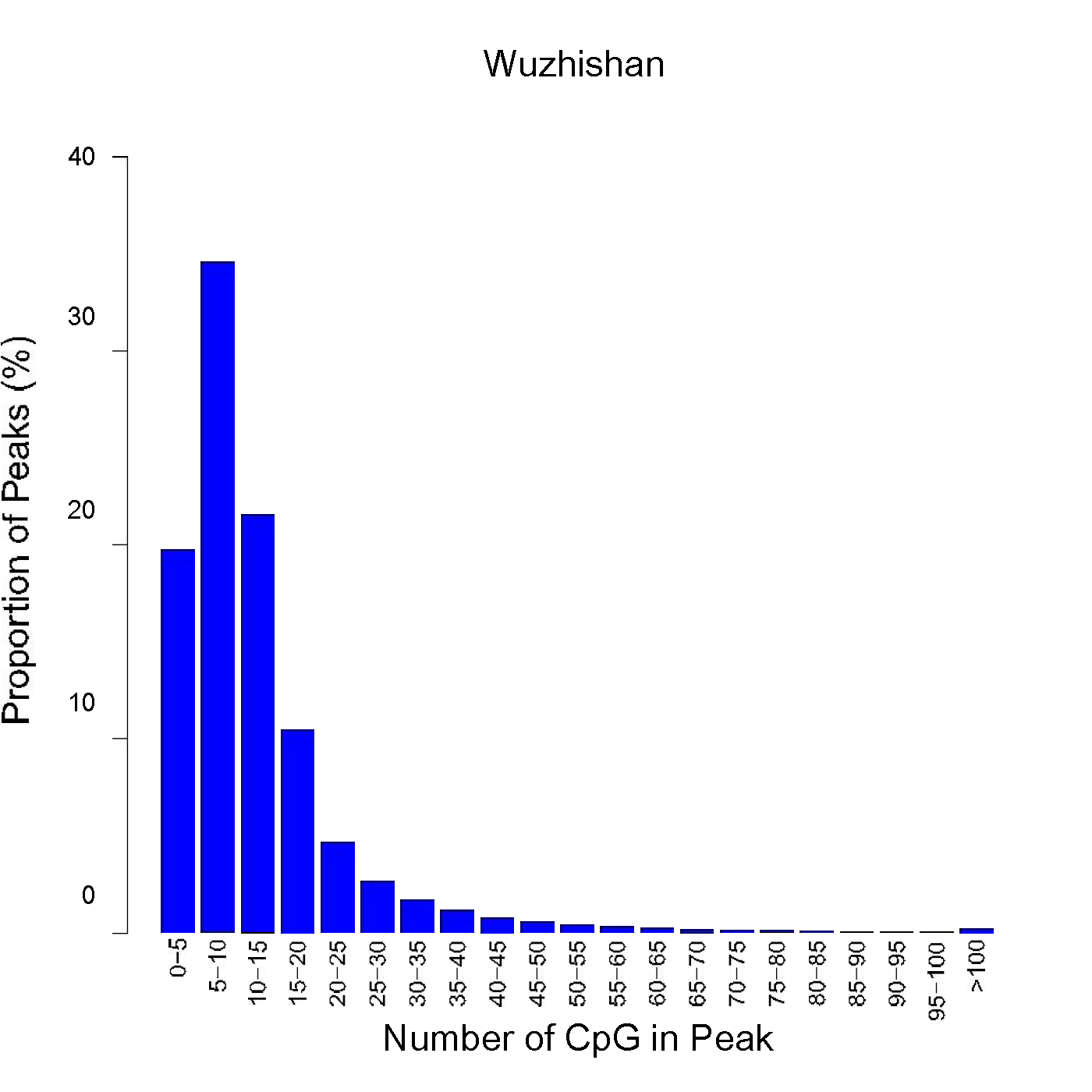


**Figure S3.** Number of CpGs in methylated peaks of Wuzhishan pigs.


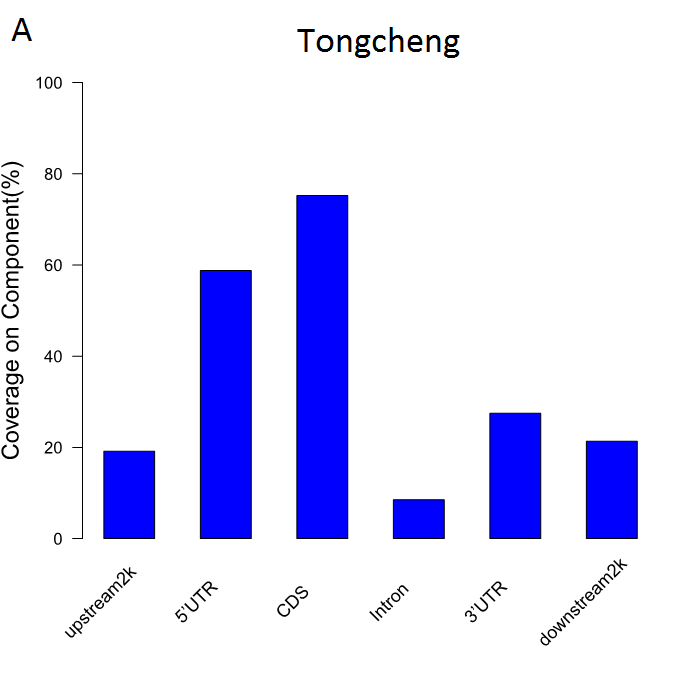

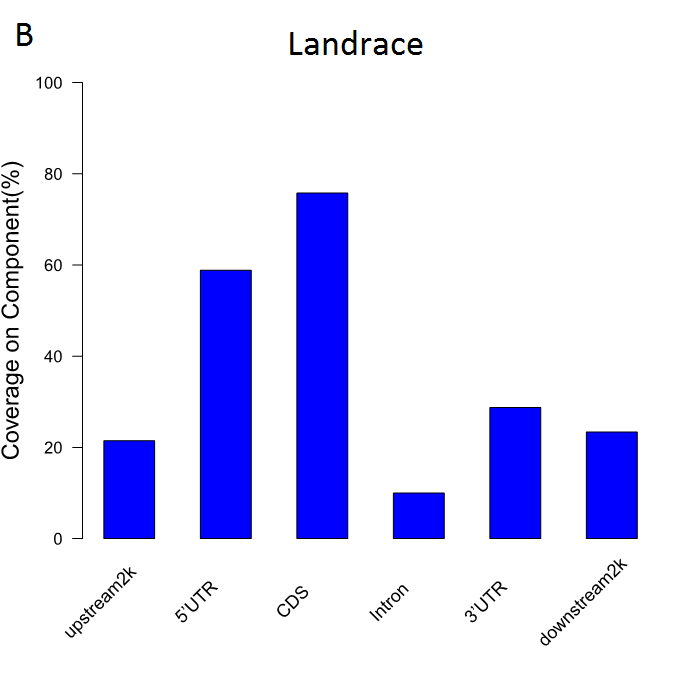


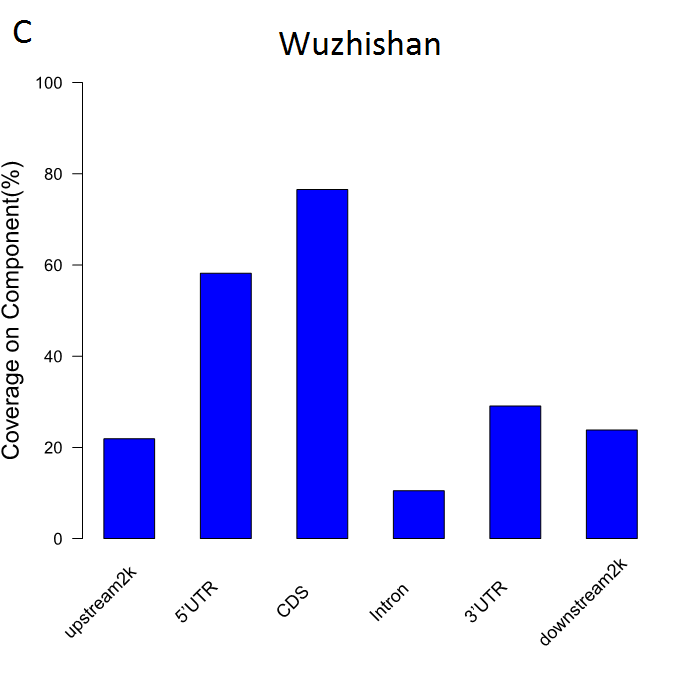


**Figure S4.** Peak genome coverage in different elements of the genome. (A) Tongcheng; (B) Landrace; (C) Wuzhishan. Genome coverage was calculated as follows: (base number of peaks in component / total base number of the component) × 100.


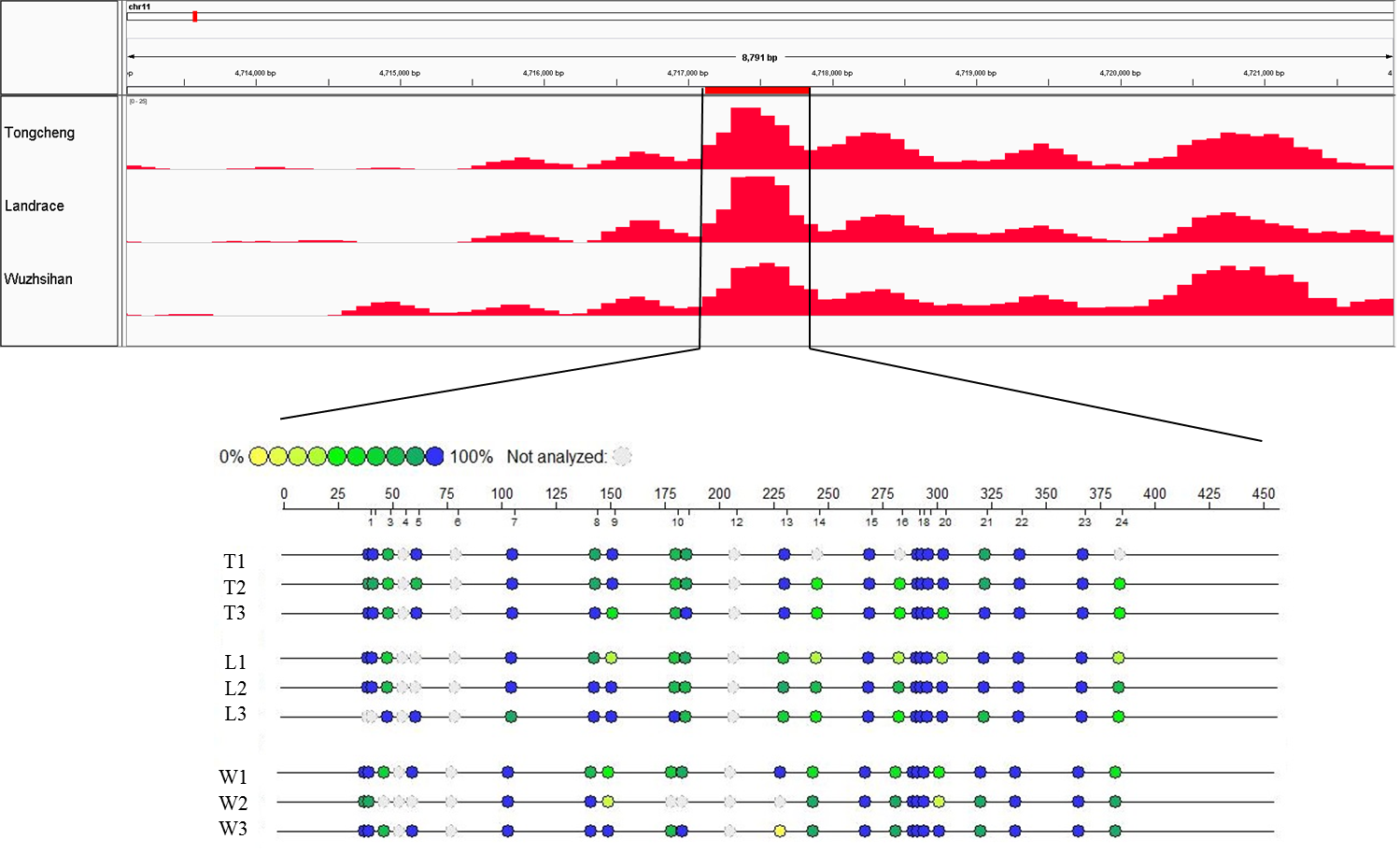


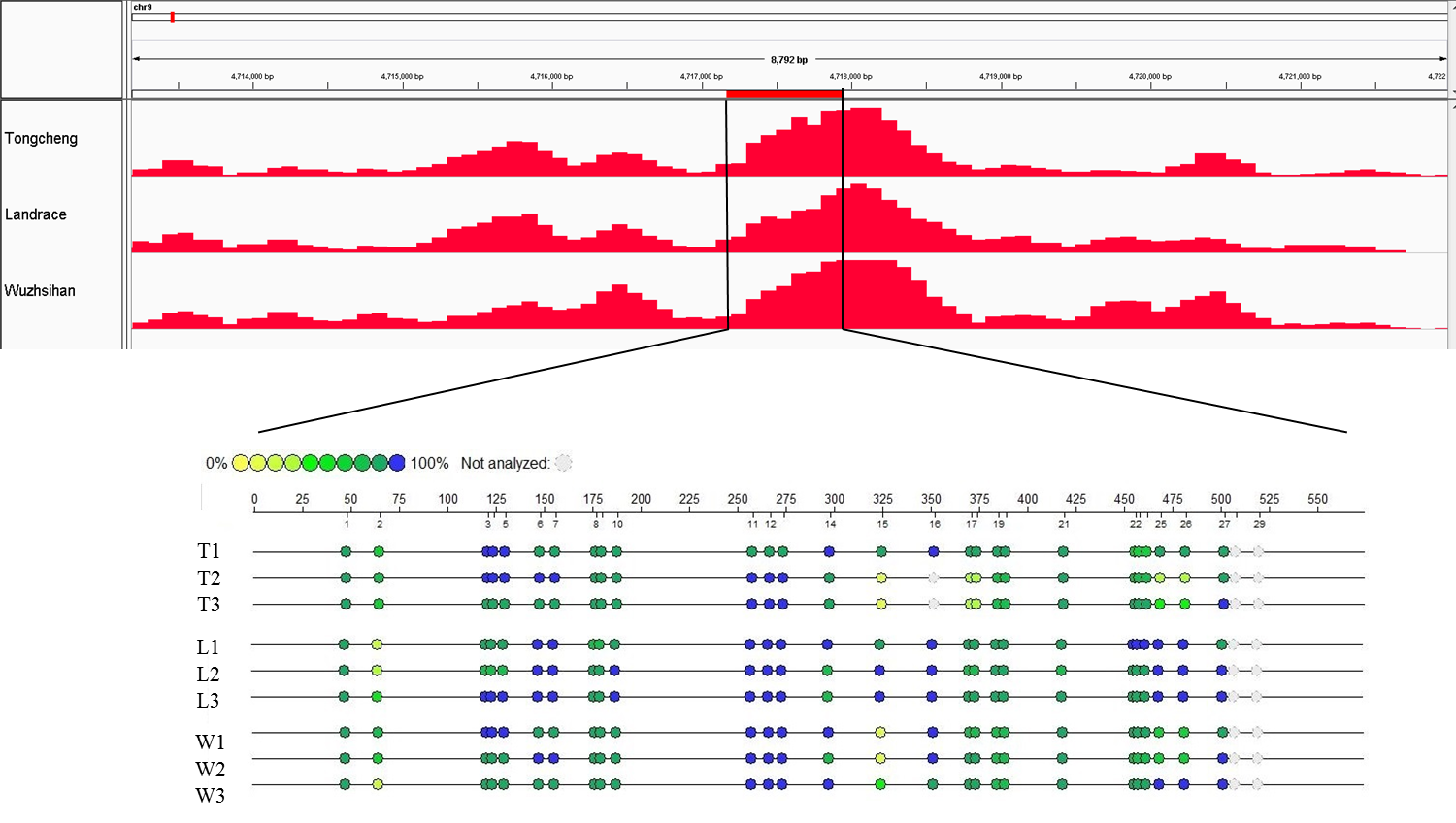


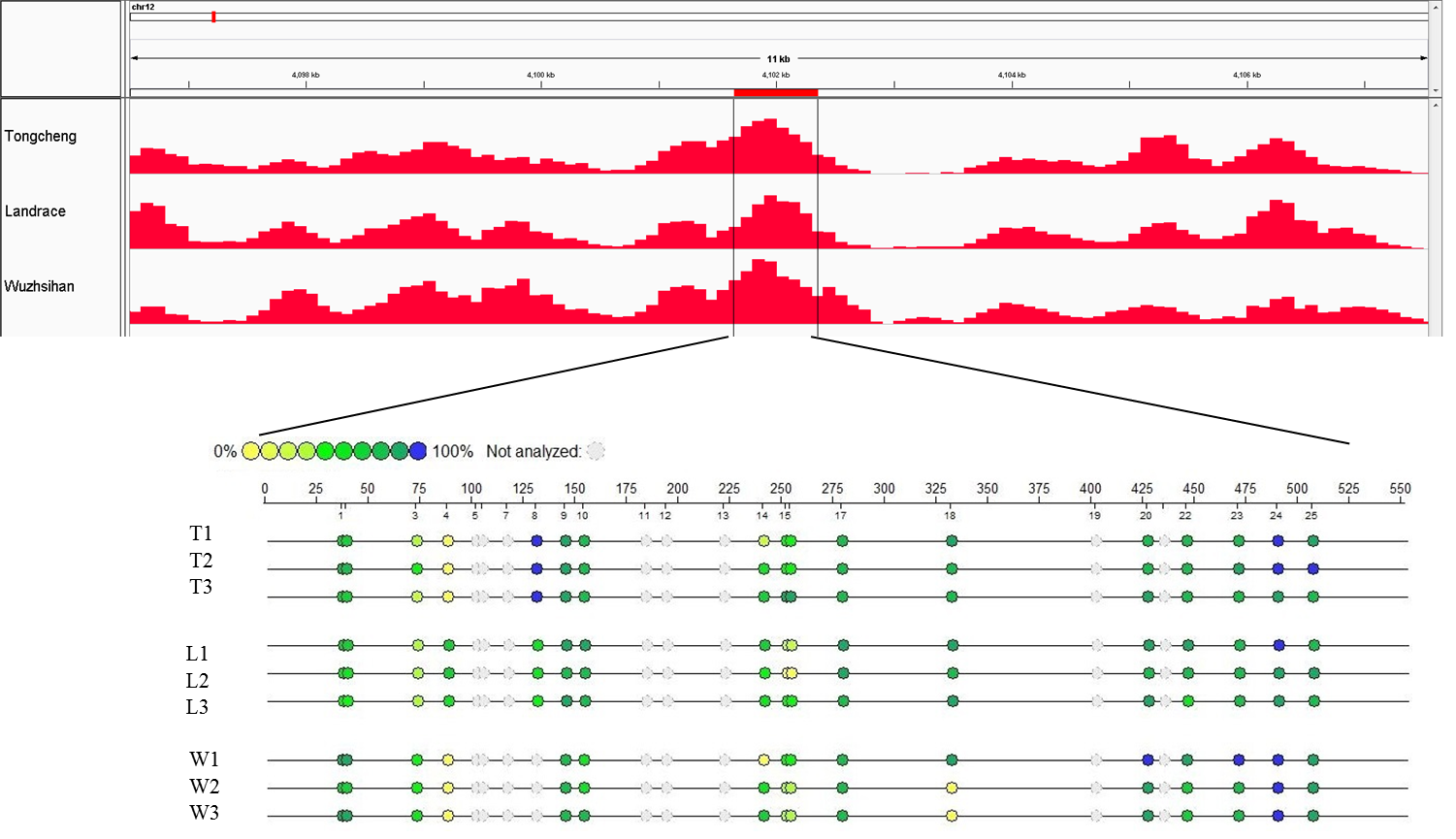


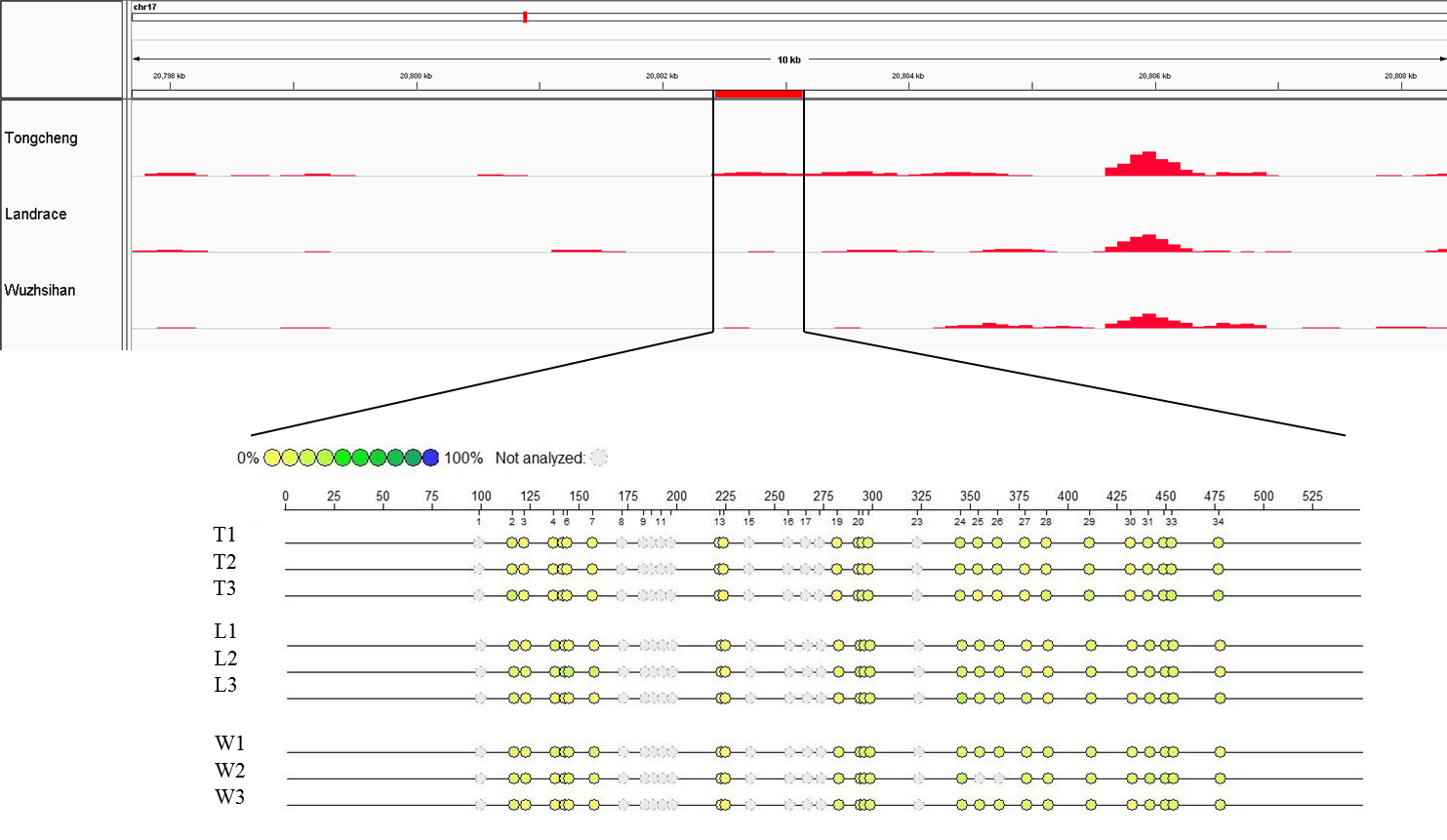


**Figure S5**. Validation of MeDIP-Seq data by quantitative MassARRAY methylation analysis in four selected regions. (A) a high methylated region on Chromosome 11 from 4,717,101 to 4717901. (B) a high methylated region on Chromosome 9 from 4,717,189 to 4,717,990. (C) a high methylated region on Chromosome 12 from 4,101,501 to 4,102,551. (D) a low methylated region on Chromosome 17 from 20,802,451 to 20,803,150. Sequencing reads generated by MeDIP-seq were viewed by Integrative Genomics Viewer and the selected regions were indicated by red bars. Methylation pattern of the selected regions was assessed by Sequenom MassARRAY. Each line represented a single strand of DNA and each circle corresponded to a single CpG dinucleotide. The blue circles indicate 100% methylation, green circles indicate around 50% methylation and yellow circles indicate 0% methylation. The results from three biological duplicate of each pig breed are shown.
